# Impacts of COVID-19 pandemic on culture-proven sepsis in neonates

**DOI:** 10.3389/fcimb.2024.1391929

**Published:** 2024-06-06

**Authors:** Xiaofeng Yang, Luxin Ren, Min Gong, Yanhong Lu, Xin Ding

**Affiliations:** ^1^ Department of Neonatology, Children’s Hospital of Soochow University, Suzhou, Jiangsu, China; ^2^ Department of Pediatrics, Richmond University Medical Center, Staten Island, NY, United States; ^3^ Department of Respiratory Medicine, Children’s Hospital of Soochow University, Suzhou, Jiangsu, China

**Keywords:** neonatal sepsis, COVID-19, epidemiology, complications, antimicrobial resistance

## Abstract

**Objective:**

To assess the effects of COVID-19 pandemic on the epidemiology of neonatal sepsis and the antibiotic resistance profiles of pathogens involved.

**Methods:**

This retrospective cohort study analyzed infants diagnosed with culture-proven sepsis at the neonatal department of a tertiary children’s hospital in East China from January 2016 to December 2022. We compared the clinical and microbiological characteristics of neonatal sepsis cases between the pre-pandemic Phase I (2016–2019) and during the COVID-19 pandemic Phase II (2020–2022).

**Results:**

A total of 507 infants with 525 sepsis episodes were included, with 343 episodes in Phase I and 182 in Phase II. The incidence of early-onset sepsis (EOS) was significantly lower during Phase II (p < 0.05). Infants in Phase II had lower gestational ages and birth weights compared to Phase I. Clinical signs such as mottled skin, severe anemia, thrombocytopenia were more prevalent in Phase II, alongside a higher incidence of complications. Notably, necrotizing enterocolitis (NEC) (p < 0.05) and meningitis (p < 0.1) occurred more frequently during Phase II. *Escherichia coli* (*E. coli*) and *Klebsiella pneumoniae* (*K. pneumoniae*) were the predominant pathogens isolated from infants of death and cases with complications. A significant decrease in the proportion of K. pneumoniae was observed in Phase II, alongside increased antibiotic resistance in both *E. coli* and *K. pneumoniae*. The period of the COVID-19 pandemic (Phase II) was identified as an independent risk factor for complications in infants with neonatal sepsis.

**Conclusion:**

COVID-19 pandemic response measures correlated with a decrease in EOS and an increase in neonatal sepsis complications and antibiotic resistance.

## Introduction

Despite advances in perinatal medicine, sepsis remains a major cause of mortality and morbidity in newborns, particularly in developing countries (Al-Matary et al., 2019). Neonatal sepsis is classified into early-onset sepsis (EOS), occurring within 72 hours of birth, and late-onset sepsis (LOS), which manifests thereafter. Factors such as premature birth, low birth weight (LBW), prolonged premature rupture of membranes, mechanical ventilation, and central venous catheter use increase the risk of sepsis in newborns (Ba-Alwi et al., 2022; Yu et al., 2022).

In response to the COVID-19 pandemic, extensive non-pharmaceutical interventions (NPIs) altered lifestyles, hospital operations, and clinical care worldwide, including travel restrictions, event cancellations, social distancing, mask mandates, and lockdowns. These measures have impacted hospital protocols, leading to tightened infection-control practices and changes in medical care delivery such as deprogramming of elective surgeries and restricting of physical visits. For instance, social distancing has been linked to reduced bacteremia, while endogenous infection cases such as hospital-diagnosed bacteremia associated with the NPIs have risen (Brueggemann et al., 2021; Cauhape et al., 2023).

The impact of the COVID-19 pandemic on neonatal intensive care units (NICUs) has been complex and multifaceted. A study conducted in an Italian NICU showed a reduction in colonization by multiresistant organisms among infants, attributed to strengthened infection-control measures post-COVID-19 outbreak (Pezzotta et al., 2021). In contrast, there was an increase in the incidence of central-line associated bloodstream infections during the pandemic, linked to shortages of alcohol-based hand rubs (Kharrat et al., 2021). Additionally, a retrospective, observational, multi-center study involving four Italian NICUs aimed to assess the indirect effects of pandemic-related measures on very-low-birthweight (VLBW) infants. Their findings indicated that the implementation of enhanced NICU hygiene policies likely reduced the occurrence of LOS in these high-risk settings (Indrio et al., 2022).

Despite China being the first country to confront COVID-19 and implement NPIs, there is a lack of reports assessing how these measures have affected neonatal sepsis. In the current study, we analyzed clinical data from two distinct periods at a tertiary pediatric hospital to compare neonatal sepsis cases before (January 2016 to December 2019, Phase I) and during the pandemic (January 2020 to December 2022, Phase II). Our aim was to assess the indirect effects of pandemic-related measures on the epidemiology and antimicrobial resistance of neonatal sepsis.

## Methods and materials

### Study design and participants

This single-center retrospective cohort study was conducted at the Neonatal Department of Children’s Hospital of Soochow University located in East China, from January 2016 to December 2022. The study period was divided into two phases, Phase I (January 2016 to December 2019) representing the pre-pandemic era, and Phase II (January 2020 to December 2022) during the COVID-19 pandemic. The Phase I group served as the baseline for comparison. Data were extracted from the hospital’s pediatric database and patient files. All neonates diagnosed with sepsis confirmed by positive blood or cerebrospinal fluid (CSF) cultures within the specified timeframe were included.

The exclusion criteria were as follows: 1) Age beyond 28 days post-birth with a corrected gestational age over 44 weeks; 2) Absence of abnormal clinical manifestations (Subspecialty Group of Neonatology, the Society of Pediatric, Chinese Medical Association and Neonatology Society, Chinese Medical Doctor Association, Professional Committee of Infectious Diseases, 2019) (details are given in **Supplementary Information**); 3) For only one positive culture of Coagulase-negative Staphylococci (CoNS) or cultured outcome suspected to be contaminated, less than two positive results were detected in two consecutive non-specific blood tests within a 24-hour interval (Subspecialty Group of Neonatology, the Society of Pediatric, Chinese Medical Association and Neonatology Society, Chinese Medical Doctor Association, Professional Committee of Infectious Diseases, 2019); 4) Presence of malignancy, congenital malformation, or autoimmune disease; 5) Incomplete or missing medical records.

The patient medical records were meticulously examined to collect vital socio-demographic data and key factors related to neonatal sepsis, including clinical symptoms, hematological parameters, pathogen types, and antimicrobial resistance.

### Definitions and clinical criteria

Small for gestational age (SGA): Birth weight (BW) below the 10^th^ percentile for gestational age (GA) and gender.

Hyperbilirubinemia: Need for phototherapy for jaundice.

Severe anemia: Hemoglobin concentration below 6mmol/L ([mg/dl] = [mmol/l] ×1.61) in full-term infants (Zonnenberg et al., 2016); and determined by need for a blood transfusion based on hemoglobin levels, days after birth, and respiratory status in premature infants (Whyte et al., 2014) (**Supplementary Table 1**).

Leucopenia: White blood count below 5,000/mm^3^.

Leukocytosis: White blood count ≥ 30,000/mm^3^ within 3 days of birth and ≥ 20,000/mm^3^ thereafter (Subspecialty Group of Neonatology, the Society of Pediatric, Chinese Medical Association and Neonatology Society, Chinese Medical Doctor Association, Professional Committee of Infectious Diseases, 2019).

Thrombocytopenia: Platelet count below 100×10^9^/L in very premature infants, and below 125×10^9^/L in late preterm or term infants (Wiedmeier et al., 2009).

Elevated liver enzymes: ALT above 80 U/L.

Death: Mortality attributed to sepsis.

### Statistical analysis

Data were analyzed using SPSS 20.0. Statistical significance was set at a two-tailed p-value of less than 0.05. Continuous variables were presented as medians with interquartile ranges. The Mann-Whitney test was used for nonparametric data comparisons. The Chi-square (χ^2^) test or Fisher’s exact test was used for categorical data. Univariate logistic regression models assessed correlations between clinical risk factors and complications. Multivariate logistic regression modeling was then performed to estimate associations between covariables and complications. Data were reported as odds ratios (OR) and 95% confidence intervals (CI).

### Ethical considerations

The study adhered to the Declaration of Helsinki’s ethical standards and received approval from the Clinical Trial Ethics Review Committee of Children’s Hospital of Soochow University (No. 2021CS111). Individual consent for this retrospective analysis was waived.

## Results

**Figure 1:**
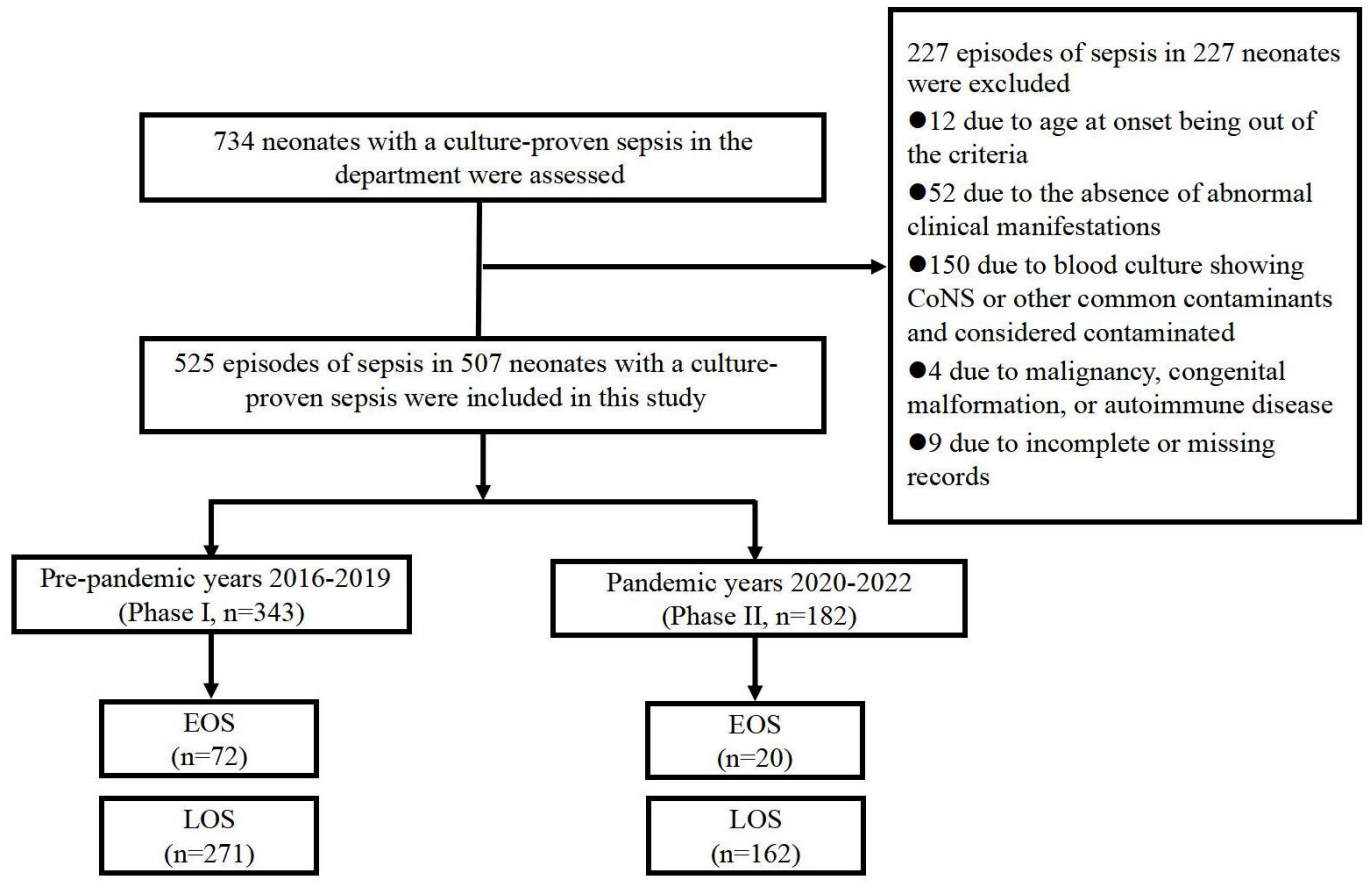
The flowchart of the number of cases included in the study.

Out of 734 neonates with culture-proven neonatal sepsis, 507 were included in the study after excluding 227 for not meeting the criteria during the study period. Of the 227 sepsis episodes in these excluded 227 neonates, the reasons for exclusion were as follows: 12 episodes were excluded due to age at onset being outside the criteria, 52 episodes were excluded because of the absence of abnormal clinical manifestations, 150 episodes were excluded because the blood culture showed only a single positive result for CoNS or other common contaminants and was considered contaminated, 4 episodes were excluded due to malignancy, congenital malformation, or autoimmune disease, and 9 episodes were excluded due to incomplete or missing records. The analysis identified 525 sepsis episodes in 507 infants, including 343 in Phase I and 182 in Phase II. EOS occurred in 92 episodes (19.4%), and LOS in 432 episodes (80.6%) (**Figure 1**). Of the 525 episodes, 443 underwent lumbar puncture (84.3%), yielding 59 positive CSF cultures (13.3%). Among these, 28 were from neonates with negative blood cultures, while the remaining 31 had matching organisms in both blood and CSF.

**Figure 2:**
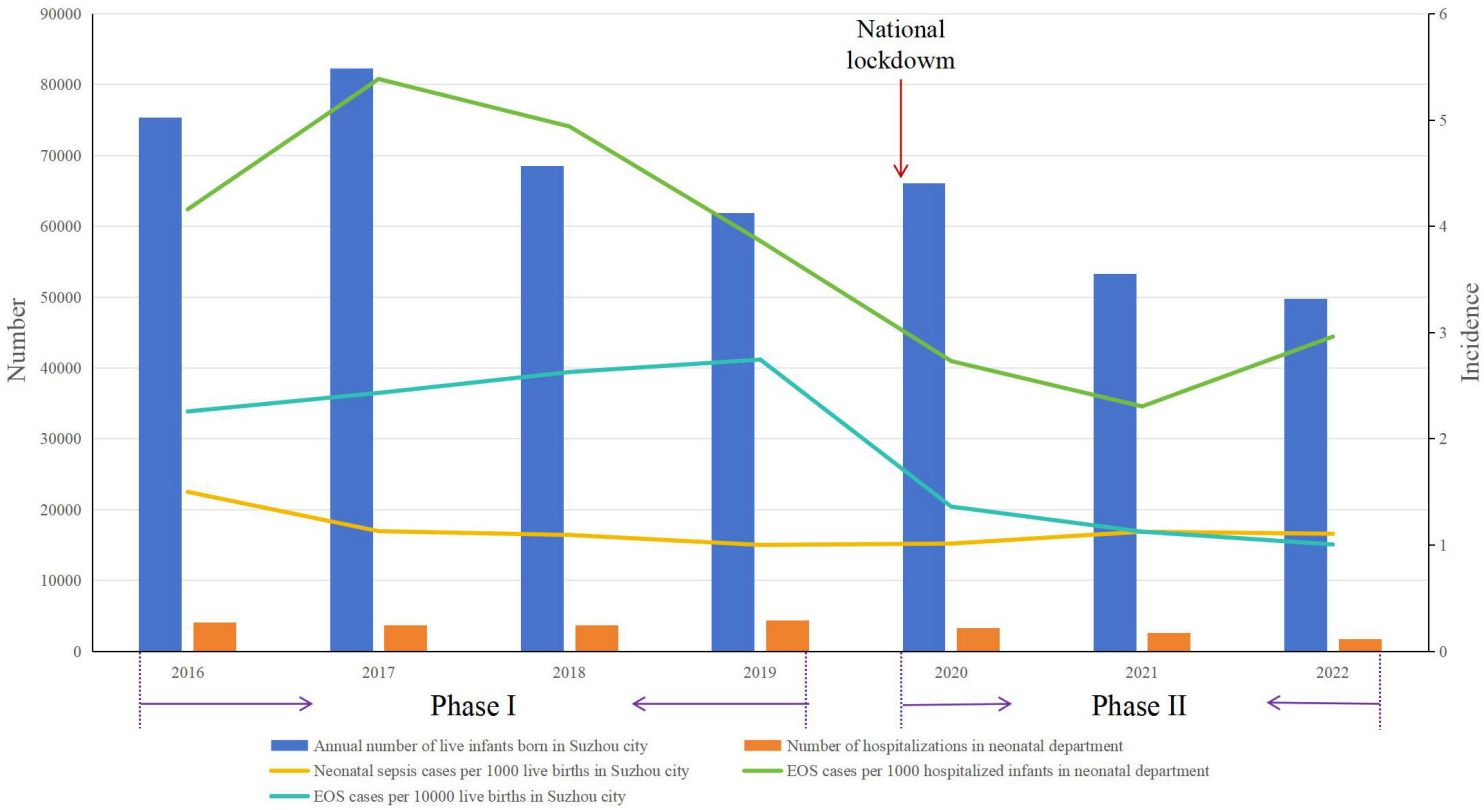
Distribution of EOS incidence during Phase I and Phase II. Phase I: pre-pandemic years (January 2016 to December 2019); Phase II: COVID-19 pandemic years (January 2020 to December 2022); EOS, early-onset sepsis.

The annual birth rate in Suzhou city has declined since 2021, rather than since 2020 when the pandemic began (**Figure 2**, https://tjj.suzhou.gov.cn/sztjj/ndjb/nav_list.shtml). Concurrently, the incidence of EOS decreased starting in 2020, calculated based on both the birth population in Suzhou city and hospitalized infants at the Neonatal Department (**Figure 2**).

### Characteristics of sepsis episodes

The median age at onset shifted from 13 days (interquartile range: 5, 22) in Phase I to 18.5 days (interquartile range: 9, 30) in Phase II, showing a significant difference (p < 0.05). Neonates in Phase II had notably smaller GAs and lower BWs than those in Phase I, and multiple gestation and maternal intrapartum fever was more common in Phase I. LOS remained as the predominant form of neonatal sepsis in both phases, with significantly different distributions of EOS and LOS (p < 0.05). There were no significant differences between the phases regarding sex, small for gestational age (SGA), membrane rupture >18 h before delivery, meconium-stained amniotic fluid, Apgar scores < 7 both at 1 min and 5 min, or the use of arteriovenous catheters or endotracheal intubation (**Table 1**).

**Table 1:** Characteristics of neonatal sepsis episodes: Phases I vs. II.

Parameter	Phase I (n = 343)	Phase II (n = 182)	p value
Age at onset, median (IQR), day	13.0 (5.0, 22.0)	18.5 (9.0, 30.0)	0.000
GA, median (IQR), week	38.4 (33.7, 39.7)	37.8 (31.7, 39.3)	0.008
BW, median (IQR), g	3150.0 (1859.0, 3500.0)	2855.0 (1711.0, 3500.0)	0.090
Male, n (%)	198 (57.7)	106 (58.2)	0.909
SGA, n (%)	31 (9.0)	13 (7.1)	0.456
Multiple gestation, n (%)	20 (5.8)	3 (1.6)	0.025
Membrane rupture >18 h before delivery	120 (35.0)	53 (29.1)	0.174
Maternal intrapartum fever	61 (17.8)	19 (10.4)	0.026
Meconium stained amniotic fluid	17 (5.0)	6 (3.3)	0.503
Apgar score at 1 min<7	29 (8.5)	18 (9.9)	0.584
Apgar score at 5 min<7	13 (3.8)	8 (4.4)	0.816
Arteriovenous catheters	74 (21.6)	48 (26.4)	0.215
Endotracheal intubation	33 (9.6)	27 (14.8)	0.074
EOS, n (%)	72 (21.0)	20 (11.0)	0.004
LOS, n (%)	271 (79.0)	162 (89.0)	0.004

GA, gestational age; BW, birth weight; SGA, smaller for gestational age; EOS, early-onset sepsis; LOS, late-onset sepsis; Phase I, Pre-pandemic years (January 2016 to December 2019); Phase II, COVID-19 pandemic years (January 2020 to December 2022).

### Clinical features and laboratory parameters

Temperature instability was a common clinical finding in both two phases. The frequency of mottled skin, severe anemia and thrombocytopenia was significantly higher during Phase II (p < 0.05). Other clinical features and laboratory parameters failed to demonstrate significant differences (p > 0.05) (**Table 2**).

**Table 2:** Comparison of clinical features and laboratory parameters: Phase I vs. Phase II.

Parameters	Phase I (n = 343) n (%)	Phase II (n = 182) n (%)	p value
Temperature instability	192 (56.0)	117 (64.3)	0.066
Poor feeding	75 (21.9)	52 (28.6)	0.088
Lethargy	95 (27.7)	66 (33.0)	0.208
Mottled skin	55 (16.0)	44 (24.2)	0.023
Feeding intolerance	48 (14.0)	37 (20.3)	0.061
Convulsion	9 (2.6)	9 (4.9)	0.164
Apnea	40 (11.7)	31 (17.0)	0.087
Need for invasive ventilatory support	34 (9.9)	25 (13.7)	0.187
Hyperbilirubinemia	57 (16.6)	20 (11.0)	0.083
Severe anemia	41 (12.0)	44 (24.2)	0.000
Leukopenia	82 (23.9)	43 (23.6)	0.943
Leukocytosis	69 (20.1)	40 (22.0)	0.617
Thrombocytopenia	52 (15.2)	42 (23.1)	0.024

Phase I: Pre-pandemic years (January 2016 to December 2019); Phase II: COVID-19 pandemic years (January 2020 to December 2022).

### Complications

The case fatality rate for neonatal sepsis was similar across both phases. However, infants in Phase II experienced a higher prevalence of complications. Notably, necrotizing enterocolitis (NEC) occurred more frequently during Phase II (p < 0.05), and there was also a higher incidence of meningitis in this phase, although the increase was not statistically significant (p < 0.1) (**Table 3**).

**Table 3:** Comparison of complications: Phase I vs. Phase II.

Parameters	Phase I (n = 343) n (%)	Phase II (n = 182) n (%)	p value
Complications	105 (30.6)	75 (41.2)	0.015
Meningitis	77 (22.4)	55 (30.2)	0.051
NEC	19 (5.5)	19 (10.4)	0.039
Pyogenic arthritis	1 (0.3)	1 (0.5)	1.000
Elevated liver enzymes	9 (2.6)	6 (3.3)	0.660
Mortality	17 (5.0)	7 (3.8)	0.562

NEC, Necrotizing enterocolitis; Phase I, Pre-pandemic years (January 2016 to December 2019); Phase II, COVID-19 pandemic years (January 2020 to December 2022).

### Etiologic pathogens

According to **Table 4**, *Escherichia coli* (*E. coli*) was the predominant pathogen in both phases. *Group B Streptococcus* (GBS) was also notable, comprising 9.2% of isolates in Phase I and 7.7% in Phase II, matched by Enterococcus at 7.7% in Phase II. A comparative analysis between the phases showed that *Klebsiella pneumoniae* (*K. pneumoniae*), which accounted for 11.7% of the isolates in Phase I, significantly declined in prevalence in Phase II (p < 0.05).

**Table 4:** Distribution of pathogens: Phases I and II.

Pathogen	Total (N = 525) n (%)	Phase I (n = 343) n (%)	Phase II (n = 182) n (%)	p value
Gram-positive	318 (60.6)	206 (60.1)	112 (61.5)	0.741
CoNS	189 (36.0)	123 (35.9)	66 (36.3)	0.927
*Streptococcus*	21 (4.0)	14 (4.1)	7 (3.8)	1.000
*S. aureus*	19 (3.6)	14 (4.1)	5 (2.7)	0.436
GBS	46 (8.8)	32 (9.3)	14 (7.7)	0.528
*L. monocytogenes*	13 (2.5)	8 (2.3)	5 (2.7)	0.774
*Enterococcus*	27 (5.1)	13 (3.8)	14 (7.7)	0.063
Gram-negative	187 (35.6)	125 (36.4)	62 (34.1)	0.588
*K. pneumoniae*	51 (9.7)	40 (11.7)	11 (6.0)	0.039
*E. coli*	98 (18.7)	64 (18.7)	34 (18.7)	0.995
*Enterobacter*	19 (3.6)	9 (2.6)	10 (5.5)	0.138
*Klebsiella oxytoca*	2 (0.4)	1 (0.3)	1 (0.5)	1.000
Fungi	20 (3.8)	12 (3.5)	8 (4.4)	0.636
*Candida albicans*	12 (2.3)	6 (1.7)	6 (3.3)	0.357
Parapsilosis	6 (1.1)	6 (1.7)	0 (0)	0.097
*Pichia guilliermondii*	2 (0.4)	0 (0)	2 (1.1)	0.120

CoNS, Coagulase-negative *Staphylococci*; *S. aureus*, Staphylococcus aureus; GBS, *Group B Streptococcus*; *L. monocytogenes*, *Listeria monocytogenes*; *K. pneumoniae*, *Klebsiella pneumoniae*; *E. coli*, *Escherichia coli*; Phase I, pre-pandemic years (January 2016 to December 2019); Phase II, COVID-19 pandemic years (January 2020 to December 2022).

**Figure 3:**
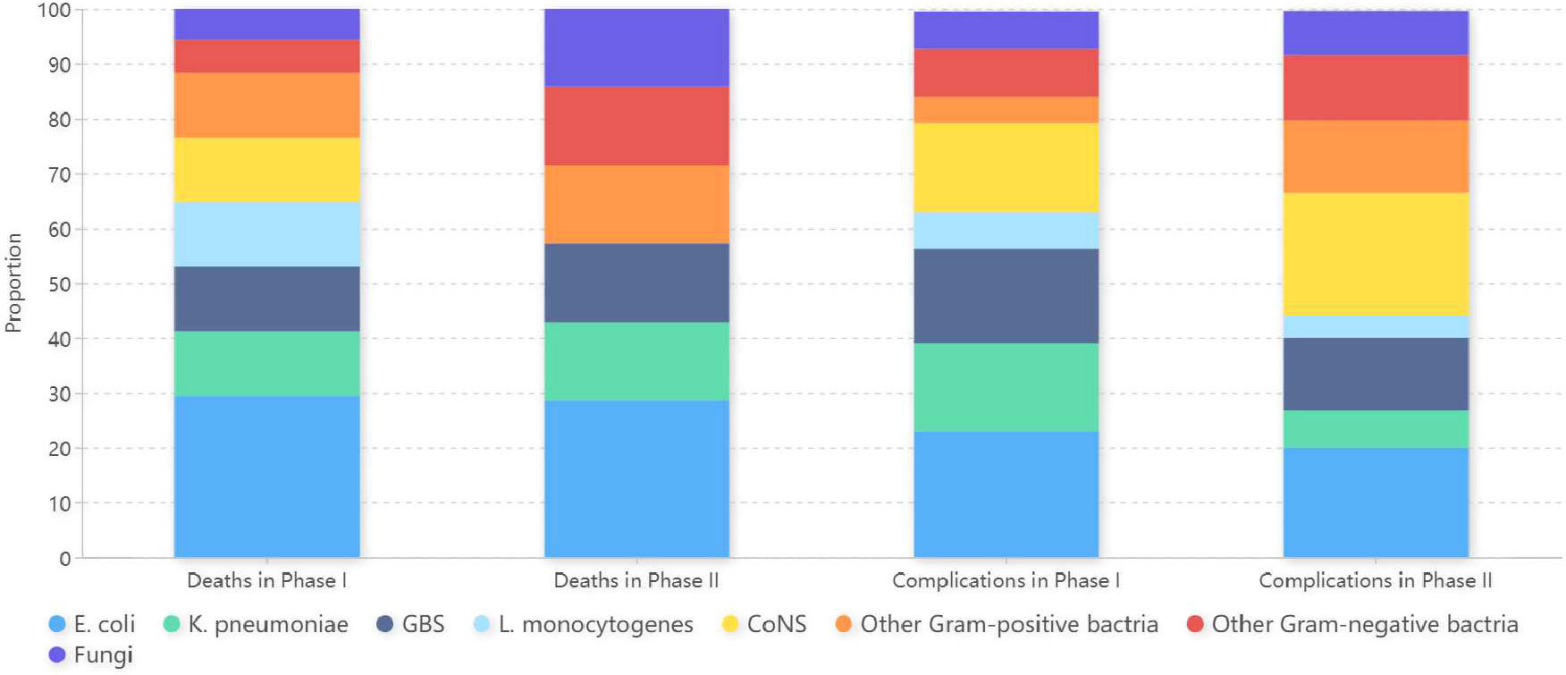
Microorganism profiles isolated from infants of death and cases with complications in Phase I and Phase II. Phase I: pre-pandemic years (January 2016 to December 2019); Phase II: COVID-19 pandemic years (January 2020 to December 2022); *E. coli*, *Escherichia coli*; *K. pneumoniae*, *Klebsiella pneumoniae*; GBS, *Group B Streptococcus*; *L. monocytogenes*, *Listeria monocytogenes*; CoNS, Coagulase-negative *Staphylococci*.

*E. coli* and *K. pneumoniae* were the most common pathogens found in infants of death and in cases with complications in both phases (**Figure 3**).

### Antimicrobial resistance

Isolates of *E. coli* and *K. pneumoniae* maintained notable susceptibility to amikacin, carbapenems, and quinolones in both Phase I and Phase II (**Tables 5**, **6**). While *E. coli* demonstrated high sensitivity to most third-generation cephalosporins, it showed significant resistance to ampicillin and cefazolin. In contrast, *K. pneumoniae* consistently exhibited low susceptibility to most cephalosporins across both phases. Furthermore, an analysis of AMR trends across the phases revealed an overall increase in resistance among the *E. coli* and *K. pneumoniae* isolates. Notably, resistance in *E. coli* to ampicillin/sulbactam and *K. pneumoniae* to piperacillin/tazobactam significantly increased in Phase II compared to Phase I (p < 0.05).

**Table 5:** Comparison of antimicrobial resistance pattern of *E. coli*: Phase I vs. Phase II.

Antimicrobial	Phase I (%)	Phase II (%)	p value
Amikacin	0/61 (0)	0/31 (0)	–
Ampicillin	48/64 (75.0)	28/34 (82.4)	0.406
Ampicillin/sulbactam	18/64 (25.0)	16/34 (47.1)	0.027
Aztreonam	9/64 (14.1)	9/34 (20.6)	0.405
Ertapenem	0/60 (0)	0/23 (0)	–
Ciprofloxacin	28/64 (43.7)	15/34 (44.1)	0.972
Piperacillin/tazobactam	2/64 (3.1)	3/34 (8.8)	0.338
Gentamicin	18/64 (28.1)	7/34 (20.6)	0.415
Cefepime	13/64 (20.3)	10/34 (29.4)	0.312
Cefuroxime	22/64 (34.4)	11/34 (32.4)	0.840
Ceftriaxone	23/64 (35.9)	12/34 (35.3)	0.950
Ceftazidime	17/64 (26.6)	9/34 (26.5)	0.992
Cefoxitin	4/64 (6.3)	2/31 (6.5)	1.000
Cefazolin	40/64 (62.5)	21/30 (70.0)	0.478
Imipenem	2/64 (3.1)	2/34 (5.9)	0.608
Sulfamethoxazole-trimethoprim	33/64 (51.6)	17/24 (70.8)	0.082
Levofloxacin	22/64 (34.4)	13/34 (38.2)	0.704

Phase I: Pre-pandemic years (January 2016 to December 2019); Phase II: COVID-19 pandemic years (January 2020 to December 2022).

**Table 6:** Comparison of antimicrobial resistance pattern of *K. pneumoniae*: Phase I vs. Phase II.

Antimicrobial	Phase I (%)	Phase II (%)	p value
Amikacin	0/36 (0)	0/9 (0)	–
Ampicillin	40/40 (100.0)	11/11 (100.0)	–
Aztreonam	24/40 (60.0)	7/11(63.6)	1.000
Ertapenem	0/30 (0)	0/6(0)	–
Ciprofloxacin	4/40 (10.0)	2/11 (18.2)	0.598
Piperacillin/tazobactam	5/40 (12.5)	5/11 (45.5)	0.015
Gentamicin	4/40 (10.0)	0/11(0)	0.565
Cefepime	19/40 (47.5)	8/11(72.7)	0.182
Cefuroxime	32/40 (80.0)	9/11(81.8)	1.000
Cefoperazone/sulbactam	23/40 (57.5)	6/11 (54.5)	0.861
Ceftriaxone	29/40 (72.5)	7/11 (63.6)	0.711
Ceftazidime	23/40 (57.5)	6/11 (54.5)	0.861
Cefoxitin	15/40 (37.5)	5/11 (45.5)	0.632
Cefazolin	34/40 (85.0)	9/11 (81.8)	1.000
Imipenem	6/40 (15.0)	4/10 (40.0)	0.097
Sulfamethoxazole-trimethoprim	22/40 (55.0)	5/11 (45.5)	0.574
Levofloxacin	1/40 (2.5)	1/11(9.1)	0.388

Phase I: Pre-pandemic years (January 2016 to December 2019); Phase II: COVID-19 pandemic years (January 2020 to December 2022).

### Associated factors for neonatal sepsis complications

**Figure 4:**
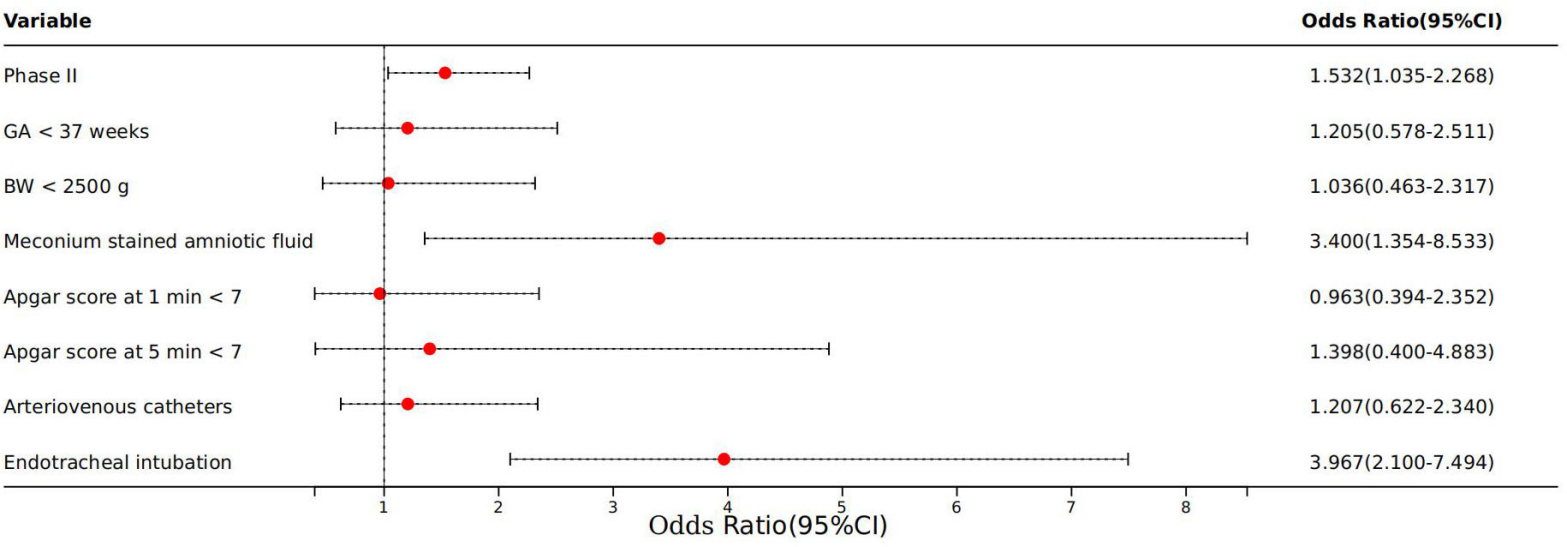
Logistic regression of complications of neonatal sepsis. Phase II: COVID-19 pandemic years (January 2020 to December 2022); GA, gestational age; BW, birth weight.

We analyzed risk factors for neonatal sepsis to determine their associations with complications in affected neonates. According to **Table 7**, eight factors demonstrated significant associations, i.e. Phase II, BW, GA, meconium-stained amniotic fluid, Apgar scores at 1 min and 5 min, use of arteriovenous catheters, and use of endotracheal intubation (p < 0.05). Further analysis using a logistic regression model included these eight significant factors. Three factors retained strong associations, including Phase II (OR = 1.532; 95% CI: 1.035–2.268; p = 0.033), meconium-stained amniotic fluid (OR = 3.400; 95% CI: 1.354–8.533; p = 0.009), and endotracheal intubation (OR = 3.967; 95% CI: 2.100–7.494; p = 0.000) (**Figure 4**).

**Table 7:** Associated factors for neonatal sepsis complications.

Parameters	Neonatal sepsis without complications (n = 345)	Neonatal sepsis with complications (n = 180)	p value
Phase II, n (%)	107 (31.0)	75 (41.7)	0.015
Age at onset, median (IQR), days	14.0 (6.0, 25.0)	15.5 (7.0, 24.0)	0.687
GA < 37weeks, n (%)	118 (34.2)	89 (49.4)	0.001
BW < 2500g, n (%)	106 (30.7)	83 (46.1)	0.000
Male, n (%)	201 (58.3)	103 (57.2)	0.819
Multiple gestation, n (%)	14 (4.1)	9 (5.0)	0.617
SGA, n (%)	31 (9.0)	13 (7.2)	0.489
EOS, n (%)	62 (18.0)	30 (16.7)	0.709
Membrane rupture > 18 h before delivery, n (%)	107 (31.0)	66 (36.7)	0.191
Maternal intrapartum fever, n (%)	52 (15.1)	28 (15.6)	0.884
Meconium stained amniotic fluid, n (%)	9 (2.6)	14 (7.8)	0.006
Apgar score at 1 min<7, n (%)	23 (6.7)	24 (13.3)	0.011
Apgar score at 5 min<7, n (%)	9 (2.6)	12 (6.7)	0.024
Arteriovenous catheters, n (%)	64 (18.6)	58 (32.2)	0.000
Endotracheal intubation, n (%)	19 (5.5)	41 (22.8)	0.000

Phase II: COVID-19 pandemic years (January 2020 to December 2022); GA, gestational age; BW, birth weight; SGA, smaller for gestational age; EOS, early-onset sepsis.

## Discussion

According to our findings, the COVID-19 pandemic and the response measures significantly altered the incidence and characteristics of neonatal sepsis at our institution, a tertiary children’s hospital in East China. The incidence of EOS showed a notable decrease during the pandemic period compared to the pre-pandemic era, a trend partially corroborated by a prior study in India (Dutta et al., 2022). However, the proportion of neonates experiencing any culture-positive sepsis, as indicated in this Indian report, demonstrated a decrease primarily attributed to a significant decline in *Acinetobacter baumannii* sepsis and sepsis caused by multi-drug resistant/extreme drug resistant/pan drug resistant organisms during the lockdown compared to the pre-lockdown period, which contrasts with our findings. EOS, typically resulting from microorganisms transmitted from mother to child before or during delivery (Schrag et al., 2016), saw a marked decrease during Phase II (2020–2022) compared to Phase I (2016–2019) in our study. This decline coincided with a reduction in maternal intrapartum fever, which may be partly attributable to the enhanced hygiene practices implemented in response to the pandemic. These measures, including maintaining social distancing, universal use of personal protective equipment, restricted patient movement and visitor policies, and rigorous cleaning protocols in the obstetrics department, likely played crucial roles. Such precautions could have reduced the transmission of infectious agents, thereby decreasing the incidence of conditions conducive to EOS. Understanding these connections is vital for developing strategies to prevent neonatal sepsis beyond the current pandemic context, suggesting that some of these enhanced hygiene practices could be beneficial if sustained in the long term.

We noted a statistically significant increase in the proportion of LOS cases from 79% (271/343) in Phase I to 89% (162/182) in Phase II. The decline in EOS may contribute to the escalation in the proportion of LOS. Furthermore, prominent risk factors for LOS include premature birth and need for intensive medical interventions such as intubation, mechanical ventilation, central catheter insertion, poor nutrition, and surgical procedures due to LBW (Pan et al., 2020; Kostlin-Gille et al., 2021). Some studies show a decrease in the premature birth rate of newborns during the COVID-19 period (Been et al., 2020; Bian et al., 2021), while conversely, other studies indicate that during the COVID-19 pandemic, the rates of premature births and low birth weight have increased (Charles et al., 2023; Gholami et al., 2023). In our findings, neonates with neonatal sepsis in Phase II presented with smaller GAs and lower BWs compared to those in Phase I, correlating with an increased proportion of LOS. Additionally, the use of endotracheal tubes also increased, though not significantly (p < 0.1). These conditions, more prevalent among preterm infants who typically require more complex care, likely contributed to the higher proportion of LOS observed during the pandemic. Indrio and his colleagues enrolled infants with VLBW and/or GA between 22 and 29 weeks from four Italian NICUs in their study. They found a decreased incidence of LOS during the pandemic period compared to the pre-pandemic period (Indrio et al., 2022), which is inconsistent with the results of our study. This discrepancy may be related to differences in GA and BW of the populations included.

In our study, clinical manifestations such as mottled skin, severe anemia and thrombocytopenia were more frequent in Phase II, coinciding with a higher rate of complications. Notably, the incidence of NEC significantly increased during this phase (p < 0.05), as did the occurrence of neonatal meningitis, though less significantly (p < 0.1). These conditions are major contributors to morbidity and mortality from neonatal sepsis, suggesting greater severity of illness during the pandemic compared to the pre-pandemic period.

Pathogen analysis revealed that CoNS, *E. coli*, *K. pneumoniae*, and GBS were the most prevalent, with *E. coli* and *K. pneumoniae* being primarily responsible for neonatal deaths and NEC cases in our center. This pathogen distribution aligns variably with global reports, reflecting regional differences in pathogen prevalence (Jiang et al., 2016; Al-Matary et al., 2019; Ba-Alwi et al., 2022). For instance, while *E. coli* and *K. pneumoniae* showed substantial susceptibility to amikacin, gentamicin, carbapenems, and quinolones in our study, research by Kumar et al (Kumar et al., 2023). indicated high resistance to amikacin and gentamicin among these pathogens. Such discrepancies can often be attributed to local antibiotic usage patterns, environmental conditions, and healthcare practices. Furthermore, our comparative analysis between phases revealed a significant decrease in the proportion of *K. pneumoniae* in Phase II. Despite this reduction, there was a marked increase in antibiotic resistance for both *E. coli* and *K. pneumoniae* during the same period.

Our analysis identified several factors associated with complications among neonatal sepsis infants, with the period of the COVID-19 pandemic (Phase II) emerging as an independent risk factor. This association was probably influenced by multiple factors, including smaller GA and lower BW observed during this period, compounded by the broader impacts of the COVID-19 epidemic and related lockdown measures.

Notably, the increased resistance of *E. coli* and *K. pneumoniae* to antibiotics during Phase II played a significant role in the complications observed, which aligns with findings from France, where a study reported an uptick in documented monomicrobial bacteremia in 2020 compared to 2019 (Cauhape et al., 2023). Another French study noted not only an increase in bloodstream infections but also a heightened incidence of infections caused by organisms resistant to third-generation cephalosporins (3GC) during the lockdown compared to the corresponding period in 2019 (Amarsy et al., 2022).

The global surge in AMR has been linked to the pandemic, as indicated by Kaba and colleagues who observed an increase in wild phenotypes of several bacterial species, including *E. coli* and *K. pneumoniae* during the lockdown periods (Kaba et al., 2021). The challenges to implement stringent antimicrobial stewardship (AS) programs during the pandemic were significant, as evidenced by a study in Ireland where 76% of respondents noted obstacles to effective AS due to COVID-19 (Martin et al., 2021).

Moreover, the pandemic led to increased prescriptions of antibiotics as a preventive measure against bacterial infections, potentially enhancing the selection pressure for resistant bacteria (Tebano et al., 2018). Concurrently, the extensive use of biocidal agents and environmental disinfectants intended to curb the spread of COVID-19 might also have contributed to the rise in AMR (Fahimipour et al., 2018; Pidot et al., 2018).

The COVID-19 pandemic has significantly disrupted neonatal care globally, particularly for the smallest and most critically ill infants (Rao et al., 2021). Breastfeeding has been reported to protect against sepsis (Carbone et al., 2020). SARS-CoV-2 RNA is found in human milk in trace amount, though WHO recommends exclusive breastfeeding in all cases unless contraindicated (Kumar et al., 2022). However, despite continued efforts to promote breastfeeding, it was less prevalent in our department during the pandemic due to stringent control measures. Therefore, there was no discussion on the effects of not breastfeeding on infants. This reduction in breastfeeding may have contributed to the increased incidence of sepsis complications.

The COVID-19 pandemic has been reported to indirectly elevate neonatal mortality within hospitals (Kc et al., 2020). Our hospital experienced about a one-third reduction in neonatal admissions, a decline partly attributable to a shift in the composition of admissions, where more patients presented with severe conditions and bacteremia. The decrease in admissions also reflected the impact of broader pandemic control measures implemented in the city, such as travel restrictions, lockdown, and the closure of public spaces, which likely delayed hospital visits until conditions worsened. This delay, combined with greater concerns about COVID-19 exposure in healthcare settings, resulted in patients presenting at more advanced stages of illness, thereby increasing the severity of complications associated with neonatal sepsis.

## Conclusions

The COVID-19 pandemic has profound impact on the epidemiology and characteristics of neonatal sepsis. Certain pandemic-related measures likely influenced hospital processes and social behaviors, contributing to a reduced rate of EOS, an increase in neonatal sepsis-associated complications and antibiotic resistance. It remains uncertain which specific interventions were most impactful, where further research is needed to isolate and analyze the factors. Future studies should focus on delineating the specific elements of pandemic response that affect neonatal health outcomes, facilitating better preparedness and response strategies in facing similar global health crises.

## Data availability statement

The original contributions presented in the study are included in the article/**Supplementary Material**. Further inquiries can be directed to the corresponding authors.

## Ethics statement

The studies involving humans were approved by Clinical Trial Ethics Review Committee of Children’s Hospital of Soochow University. The studies were conducted in accordance with the local legislation and institutional requirements. The ethics committee/institutional review board waived the requirement of written informed consent for participation from the participants or the participants’ legal guardians/next of kin because This is a retrospective clinical study.

## Author contributions

XY: Data curation, Formal analysis, Funding acquisition, Investigation, Methodology, Project administration, Resources, Software, Validation, Visualization, Writing – original draft, Writing – review & editing. LR: Conceptualization, Data curation, Investigation, Methodology, Project administration, Resources, Supervision, Validation, Visualization, Writing – original draft, Writing – review & editing. MG: Data curation, Methodology, Writing – review & editing. YL: Conceptualization, Data curation, Formal analysis, Funding acquisition, Investigation, Software, Visualization, Writing – original draft, Writing – review & editing. XD: Conceptualization, Data curation, Formal analysis, Funding acquisition, Investigation, Methodology, Software, Supervision, Writing – original draft, Writing – review & editing.
