# Supplementary material for: Impacts of COVID-19 pandemic on culture-proven sepsis in neonates
Source: Front Cell Infect Microbiol. 2024 Jun 6;14:1391929. doi: 10.3389/fcimb.2024.1391929 (PMC11186981; doi:10.3389/fcimb.2024.1391929)
Supplement: Supplementary file 1 [file DataSheet_1.docx]

Supplementary Material

Supplementary information: abnormal clinical manifestations of neonatal sepsis, as outlined by the Subspecialty Group of Neonatology and Professional Committee of Infectious Diseases in 2019, includes:

1. General symptoms

Hyper- or hypothermia, or temperature instability; poor response to stimuli, poor feeding, edema, low Apgar score.

1. Digestive system

Jaundice, abdominal distension, vomiting or gastric retention, diarrhea, hepatosplenomegaly.

1. Respiratory system

Dyspnea and apnea, cyanosis, etc. Increased oxygen requirement, or need for ventilatory support.

1. Circulatory system

Pallor, cold extremities, tachycardia, bradycardia, skin and subcutaneous lesions, such as petechial rash or sclerema; hypotension or capillary filling time > 3 seconds.

1. Urinary system

Reduced urinary output, kidney failure.

1. Hematologic System

Bleeding tendencies, purpura.

Supplementary Table 1. Hemoglobin levels for severe anemia in premature infants

| Postnatal age | Respiratory support* | No respiratory support* |
| --- | --- | --- |
| Week 1 | 115 | 100 |
| Week 2 | 100 | 85 |
| Week 3 and older | 85 | 75 |

Data presented as hemoglobin, g/L . *Respiratory support is defined as an inspired oxygen requirement in excess of 25% or the need for mechanical increase in airway pressure .
